# Bilateral synchronous salivary gland tumors: report of three cases

**DOI:** 10.1186/s13000-025-01672-9

**Published:** 2025-06-13

**Authors:** Jacqueline E. van der Wal, Mustafa Barre Magan, Lennart Flygare, Karin Nylander

**Affiliations:** 1 Department of Medical Biosciences/Pathology, Umeå University, Building 6 M, 2nd Floor,, Umeå, SE– 90185 Sweden; 2 Department of Clinical Sciences, Umeå University, Umeå, Sweden; 3 Department of Diagnostics and Intervention, Umeå University, Umeå, Sweden

**Keywords:** Salivary gland tumor, Pleomorphic adenoma, Acinic cell carcinoma, Salivary duct carcinoma

## Abstract

**Background:**

Bilateral salivary gland tumors, both benign and malignant and synchronous or metachronous are very rare.

**Case presentation:**

Here three cases of synchronous bilateral salivary gland tumors are described and discussed. Recognizing the entity is important for diagnostics and treatment planning. The first patient was a 56-year-old female with a bilateral parotid tumor, a malignant tumor, salivary duct carcinoma on the right side and a benign tumor, pleomorphic adenoma on the left side. The second patient was a 50-year old female with a bilateral benign parotid tumor, a pleomorphic adenoma. The third patient was a 51-year old female with a bilateral malignant tumor, an acinic cell carcinoma. Details on the diagnostic work-up, histopathology and treatment are described and discussed.

**Conclusions:**

In the case of a unilateral salivary gland tumor, especially of the major glands, the contralateral gland is always included in the clinical and radiological (MRI) head and neck evaluation prior to surgery, to detect or exclude possible bilateral occurrence.

## Introduction

Approximately 80% of all salivary gland tumors occur in the parotid gland. Malignant salivary gland tumors are rare, accounting for approximately 3% of all head and neck tumors [1]. Multiple (uni- or bilateral) salivary gland tumors are even rarer. These multiple salivary gland tumors, both uni-and bilateral, are also located mostly in the parotid gland, with an incidence of 0.1–3.4% [2]. Still, recognizing the entity is important for diagnostics and treatment planning. In the case of a unilateral salivary gland tumor, especially of the major glands, the contralateral gland is always included in the clinical and radiological (MRI) head and neck evaluation prior to surgery, to detect or exclude possible bilateral occurrence.

According to chronology, synchronous tumors occur at the same time but are distinct from each other either in the same gland or ipsi- or bilaterally in different glands [3, 4]. In contrast, metachronous occurrence of salivary gland tumors has been reported, indicating consecutive development after a certain time. The time frame between the first and the second tumor has not been reported in the literature and therefore can only be described as not “being present at the time of diagnosis/treatment of the first tumor” [5, 6]. Both benign and malignant tumors can present as bilateral or synchronous tumors. Postoperative recurrences and metastases are excluded from the definition of multiple salivary gland tumors [2].

Here three cases of benign and malignant synchronous bilateral salivary gland tumors, all of which were diagnosed between 2022 and 2023, are described and discussed, showing the importance of recognizing of this entity.

### Case reports

#### Patient 1

A 56-year-old nonsmoking woman sought care from a doctor due to muscle weakness in the right lower lip. On examination mild paralysis of the marginal mandibular branch of the right facial nerve was found. Stroke investigations, including Computed Tomography (CT) angiography revealed an 18 mm mass with radiological features suggestive of malignancy in the upper lobe of the right parotid gland, which was in agreement with the patient’s symptoms. No cerebrovascular disease was found.

Further investigation with magnetic resonance imaging (MRI) revealed a 2 × 1 × 3 cm mass with irregular borders in the deep lobe of the right parotid gland.

**Fig. 1:**
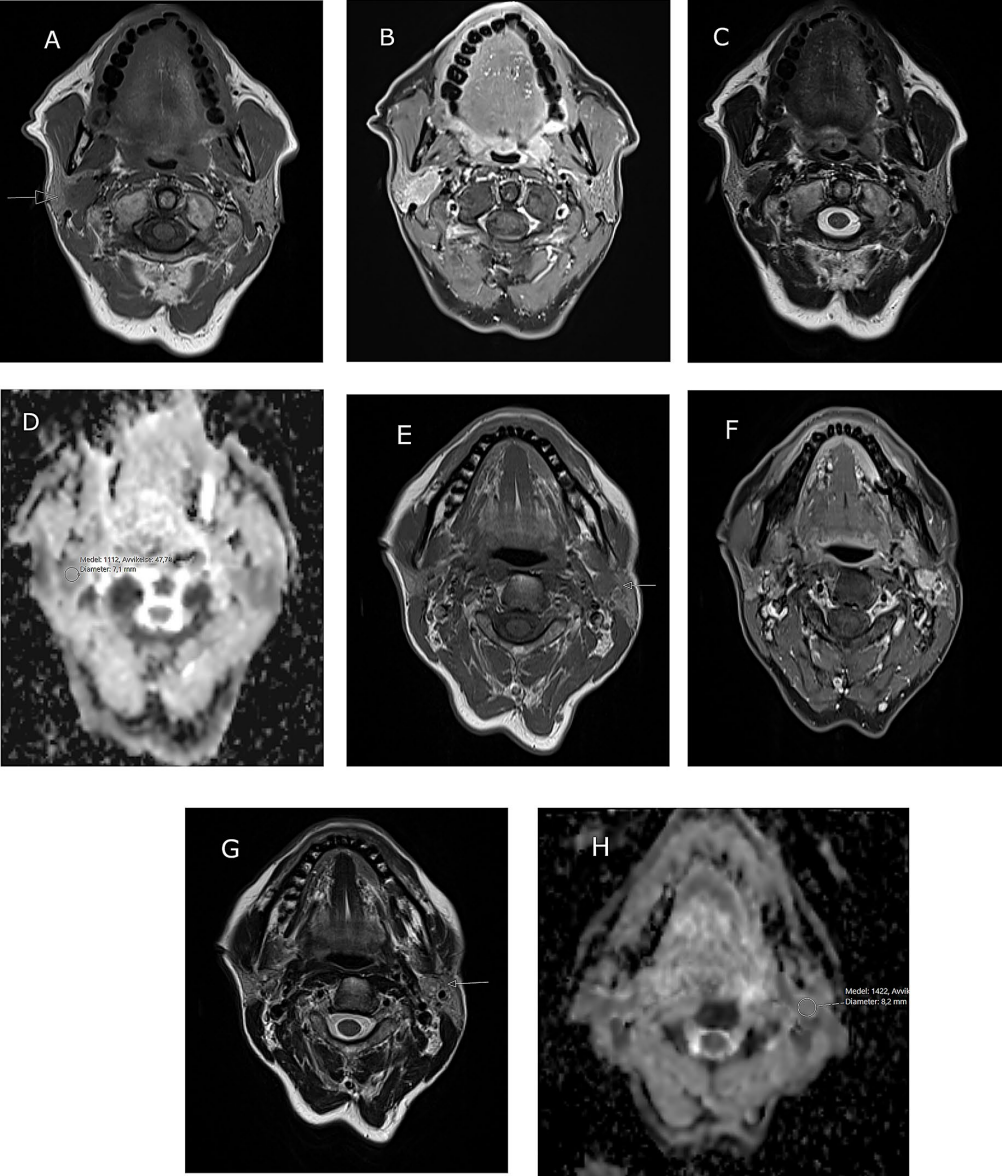
Patient 1, MR image of bilateral salivary gland tumors in a 56-year old female. **A**-**D** show a right sided intraparotid lesion with irregular borders. **A**) Low signal on T1W (arrow), **B**) rich contrast enhancement on fat-suppressed TIW, **C**) low signal on T2W and **D**) restricted diffusion on the ADC-map, ADC = 1,11 × 10^− 3^ mm^2^/s. E-H Left sided intraparotid lesion in the deep lobe. **E**) Low signal on T1W (arrow), **F**) rich contrast enhancement on fat-suppressed TIW, **G**) mixed signal on T2W (arrow) and **H**) moderate diffusion restriction on the ADC-map, ADC = 1,42 × 10^− 3^ mm^2^/s

The mass presented a low signal on T2-weighted (T2W) images, avid uptake of gadolinium-contrast (Gd) and restricted diffusion, ADC = 1.1 × 10^− 3^ mm^2^/s suggesting malignancy (Fig. 1).

The lateral aspect of the tumor was close to the expected course of the facial nerve main trunk. On the left side, MRI image revealed a 1 cm deep-lobe mass with defined borders. On T2W images a mixed signal was seen and moderately restricted diffusion, ADC = 1.4 × 10^− 3^ mm^2^/s indicating a benign lesion (Fig. 1). No pathological lymph nodes were detected. Fine-needle aspiration cytology (FNAC) was performed on the right parotid lesion, revealing a SUMP (salivary gland neoplasm of uncertain malignant potential), Milan-classification IV. The differential diagnosis included a cell-rich pleomorphic adenoma or a benign (myoepithelioma) or malignant (myoepithelial carcinoma) myoepithelial tumor; no material was available for immunohistochemical investigation. FNAC of the left parotid lesion revealed a partly degenerated and blood rich background with cells with a large amount of cytoplasm without clear atypia; the material was not suitable to make a certain diagnosis or give a differential, Milan classification I (non-diagnostic).

The tumor was clinically classified as cT3N0M0 on the right side and cT1N0M0 on the left side, according to the 8th edition of the UICC TNM classification of malignant tumors.

Surgery was performed on the right side first, followed by surgery on the left parotid 8 days later. The surgical treatment was performed in two stages due to the uncertainty surrounding the cytological diagnosis (SUMP). On the right side, subtotal parotidectomy was performed with resection of the tumor due to tumour invasion, the marginal mandibular and cervical branches of the facial nerve. Since an enlarged lymph node was observed inferior to the parotid tail, neck dissection of level 2 A was performed.

On the left side a subtotal resection of the deep lobe of the parotid gland was performed together with level 2 A lymph nodes.

**Fig. 2:**
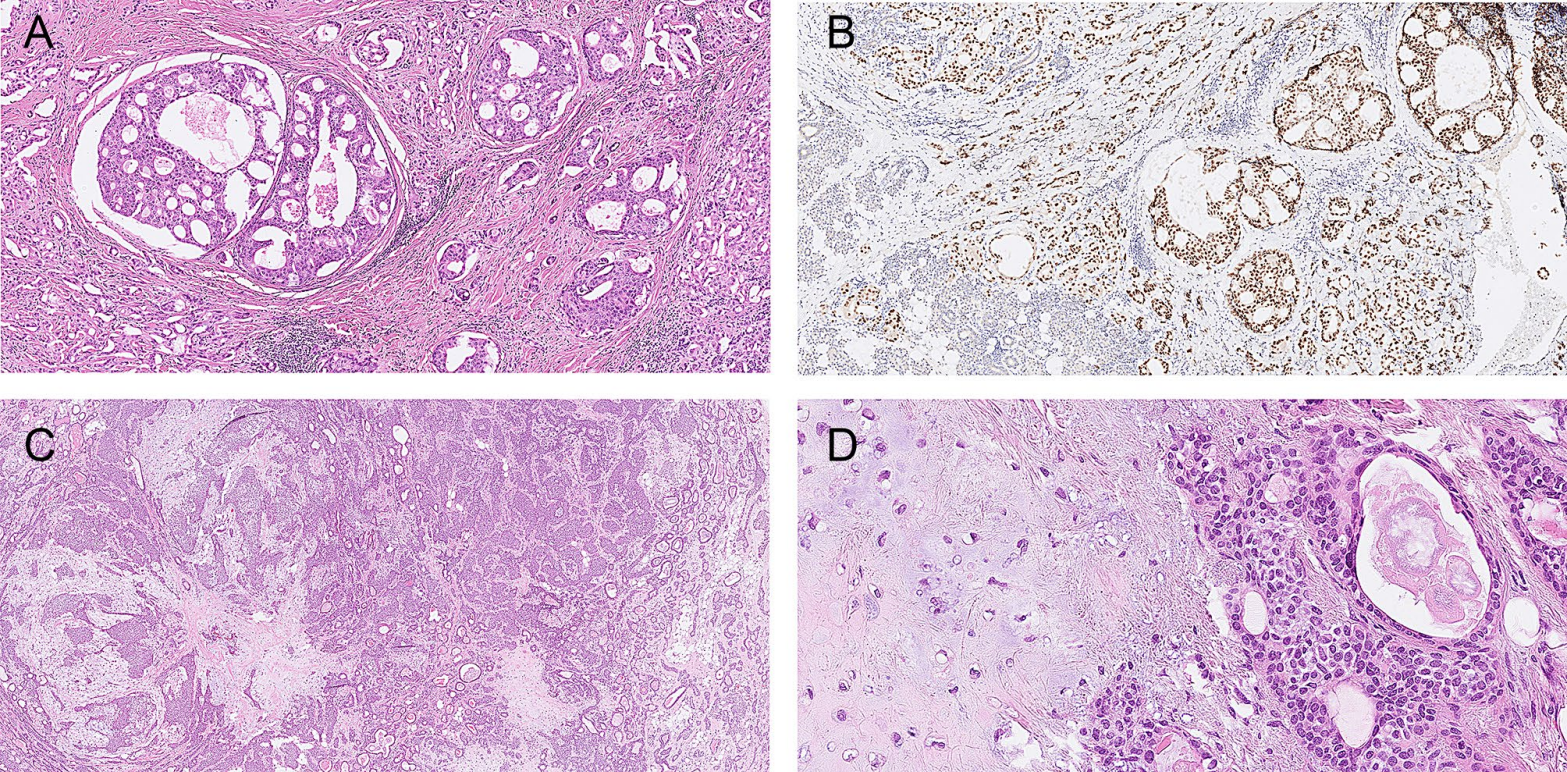
Patient 1, **A**. salivary duct carcinoma, H&E; **B** salivary duct carcinoma, androgen receptor; **C**,**D**. pleomorphic adenoma, H&E;

Microscopy of the right parotid tumor revealed an invasive tumor consisting of large cells with eosinophilic cytoplasm and enlarged nuclei with prominent nucleoli forming solid fields, ductal structures and small strands within a fibrotic background (Fig. 2). The ductal epithelium exhibited fenestrations resembling ‘Roman bridge’ architecture. Perineural invasion and lymphovascular invasion were present, just as were multiple positive lymph nodes with extracapsular extension in the parotid and level 2 A. Tumor cells were positive for CK AE1/3 (cytoplasmic), CK7 (cytoplasmic), GP15 (cytoplasmic and/or membranous) and androgen receptor (nuclear); Her2 staining (membranous) was heterogeneous, with mainly score 2+, focally 3. P63 and p40 were mostly negative. Only focally p63 positive cells were present surrounding tumorous ductal structures (intraductal areas). No signs of a pre-existing pleomorphic adenoma were present, like f.e. biphasic growth pattern, chondromyxoid background, which excludes a salivary duct carcinoma ex pleomorphic adenoma. On the basis of morphology and immunohistochemistry, a diagnosis of salivary duct carcinoma was made, and classified as pT4N2M0.

In the left parotid gland, a demarcated and partly encapsulated tumor was observed. It had a somewhat bosselated margin and consisted of a chondromyxoid background in which small and larger anastomosing fields, strands and ducts of small cells with little eosinophilic cytoplasm and small nuclei were observed. There was no atypia or invasive growth. Since the histomorphological aspects of the tumor supported a diagnosis of pleomorphic adenoma no additional (immunohistochemical) stainings were needed.

On the right side postoperative radiotherapy was given; before this started a lymph node in the parotid area appeared, indicating growth of the salivary duct carcinoma. No tumor occurred during recent follow-up.

In accordance with the Swedish national guidelines for head and neck cancer, patients are monitored every three months the first two years, and from years three to five follow-up is scheduled every six months. For certain patient groups, such as those with adenoid cystic carcinoma, extended follow-up of up to 10 years is typically recommended.

A post-therapeutic baseline MRI is generally performed at the initial follow up, followed by additional MRI scans in cases of clinical deterioration, with a supplementary MRI examination often at the final tumour check.

Chemotherapy and other medical oncological treatments as adjuvant therapy for major salivary gland carcinomas are in Sweden primarily used for palliative purpose.

#### Patient 2

A 50-year-old nonsmoking woman had bilateral nodules in the parotid glands for 1.5 years. Recently, mild pain had appeared in the left parotid. The masses were both clinically localized preauricularly in the parotid gland and palpated as small sized (clinically 10–15 mm on the left side and 5–7 mm on the right side), solid, nontender, mobile nodules.

The medical history revealed that at the age of 13, a tumor had been removed from the right submandibular gland with an uncertain diagnosis, however, no further information was present on this lesion.

**Fig. 3:**
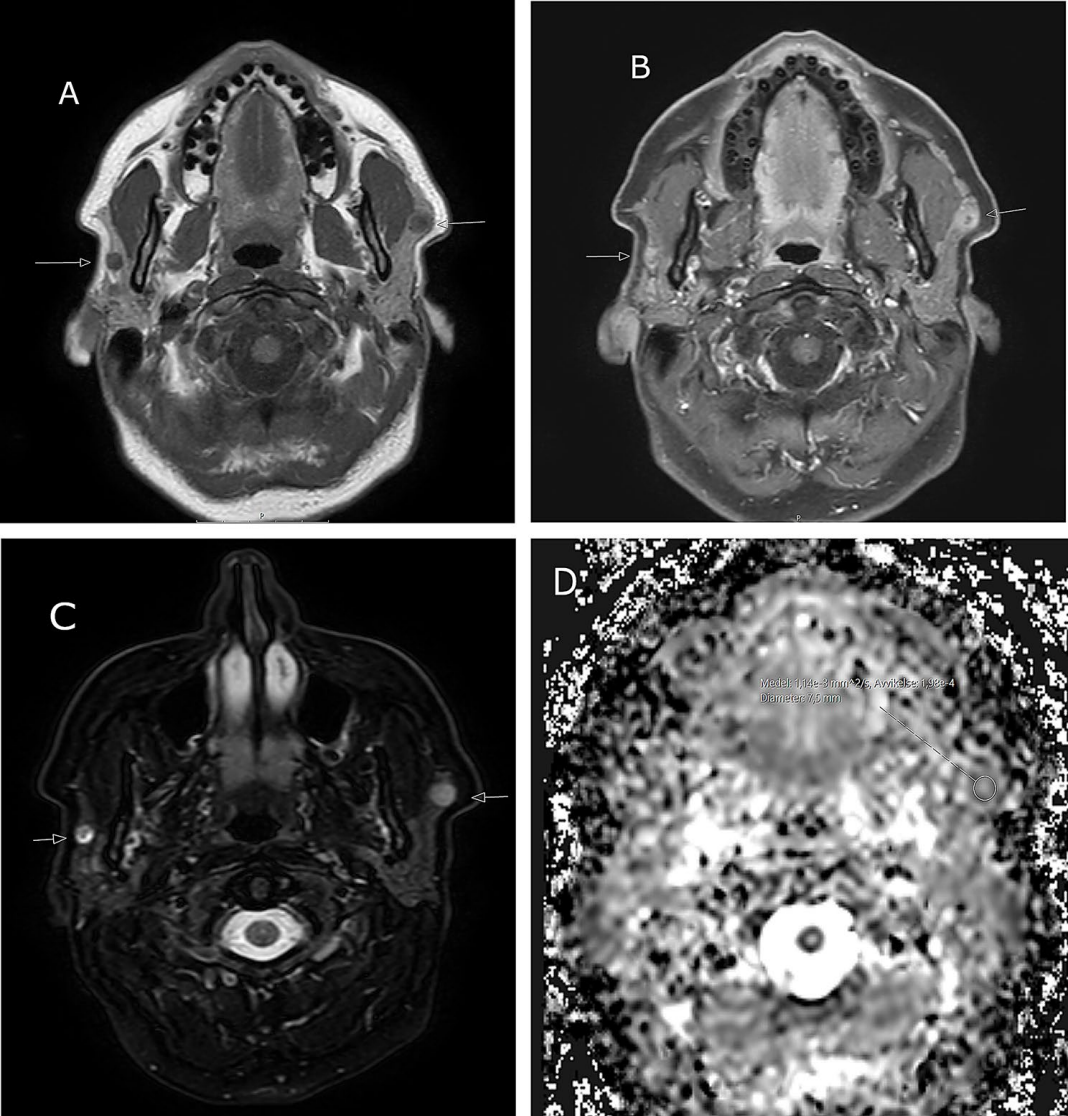
Patient 2, MR image of bilateral salivary gland tumours in a 50-year old female. **A**-**D** Demonstration of bilateral intraparotid lesions in the superficial portions. **A**) Low signal on T1W (arrows), **B**) rich, irregular contrast enhancement on fat-suppressed TIW (arrows), **C**) high signal on fat-suppressed T2W (arrows) and **D**) restricted diffusion of the left sided lesion on the ADC-map, ADC = 1,14 × 10^− 3^ mm^2^/s

MRI demonstrated bilateral nodules in the cranial part of the superficial portion of the parotid. The lesions were well-defined and rounded with mixed signals on T2W images. Diffusion was restricted on the left side, and the Apparent Diffusion Coefficient (ADC) was approximately 1.2 × 10^− 3^ mm^2^/s (Fig. 3). Dynamic contrast enhanced MRI (DCE-MR) revealed avid contrast enhancement with a fast wash-in followed by a plateau phase with minimal wash-out, and a Time Intenstity curve (TIC) type C [7]. These TICs are usually indicative of malignant lesions but have also been reported to occur in pleomorphic adenomas with high cellularity [7].

FNAC on both sides was performed and showed ductal cells, fat cells and pre-existing acini within a background of blood. Cell blocks were performed showing ductal structures without atypia. Immunohistochemistry showed positivity for AE1/3 (cytoplasmic), CK7 (cytoplasmic, inner ductal cells), p63 (nuclear, outer myoepithelial cells), S100 (cytoplasmic, outer myoepithelial cells). Due to the mostly biphasic architecture of small ductal structures, together with the radiological findings (TICs) a differential of a cell rich pleomorphic adenoma or an epithelial-myoepithelial carcinoma was made. Other biphasic salivary gland tumors were considered but seemed less likely due to the cellular and architectural morphology of the tumors.

Bilateral superficial parotidectomy was performed without any complications.

**Fig. 4:**
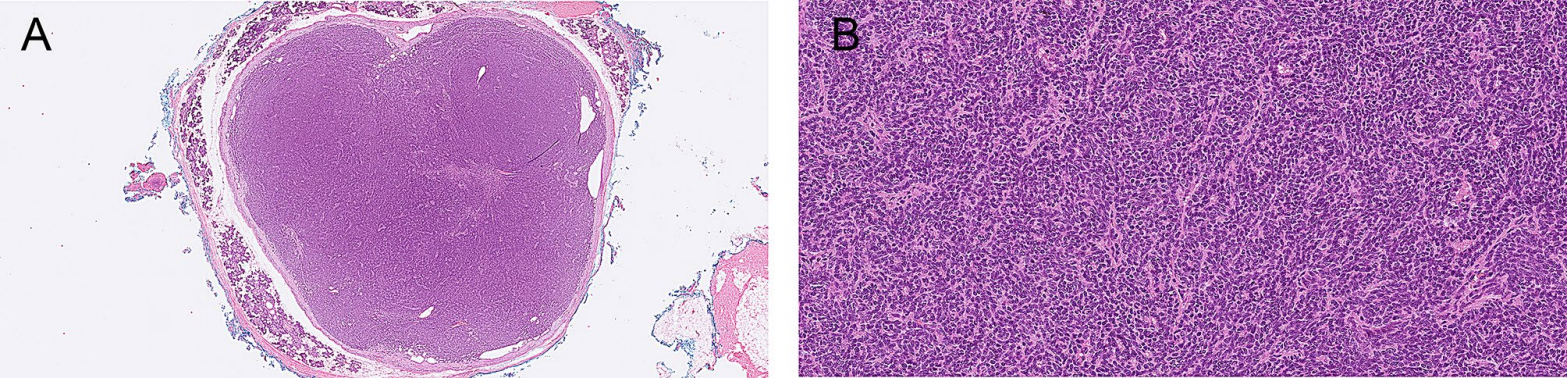
Patient 2, **A** and **B** pleomorphic adenoma on the left side, H&E. Both pleomorphic adenomas had the same morphology

Macroscopically, an encapsulated tumor with a maximum diameter of 1.6 cm was observed in the left parotid gland (Fig. 4). An intact capsule was confirmed microscopically. The tumor consisted mainly of glandular structures with a biphasic morphology of inner ductal cells (CK5, CK7 positive) and outer myoepithelial cells (p63 and S100 positive). No clear cell myoepithelial cells were found. Focally, some myxoid aspects were present. No atypia. The proliferation rate (Ki-67) varied between 2 and 20% within the tumor.

Due to the mainly biphasic structure of the tumor in addition to a pleomorphic adenoma, an epithelial-myoepithelial carcinoma was considered. Molecular analysis (NGS-analysis with Archer FusionPlex and Archer VariantPlex) was performed, due to the differential, especially looking for *PLAG1* and *HRAS*-mutations. However, no genetic aberrations were detected.

In the right parotid gland a tumor with exactly the same morphology and immunohistochemical profile was present, with a proliferation-rate (Ki-67) varying between 1.5 and 11%.

Due to the demarcation of both tumors and the absence of true invasion and atypia, the final diagnosis was bilateral pleomorphic adenoma, with a focally increased proliferation rate.

The same follow up schedule as described under patient 1 was applied. No tumor occurred during recent follow-up.

#### Patient 3

A 51-year-old smoking woman had a fluctuating resistance in the left parotid gland for approximately 7–8 years. She suffered from first bite syndrome (mouth pain when salivating or taking the first few bites of a meal). When the patient sought medical care for her complaints, the same kind of lesion was also noticed in the right parotid gland.

**Fig. 5:**
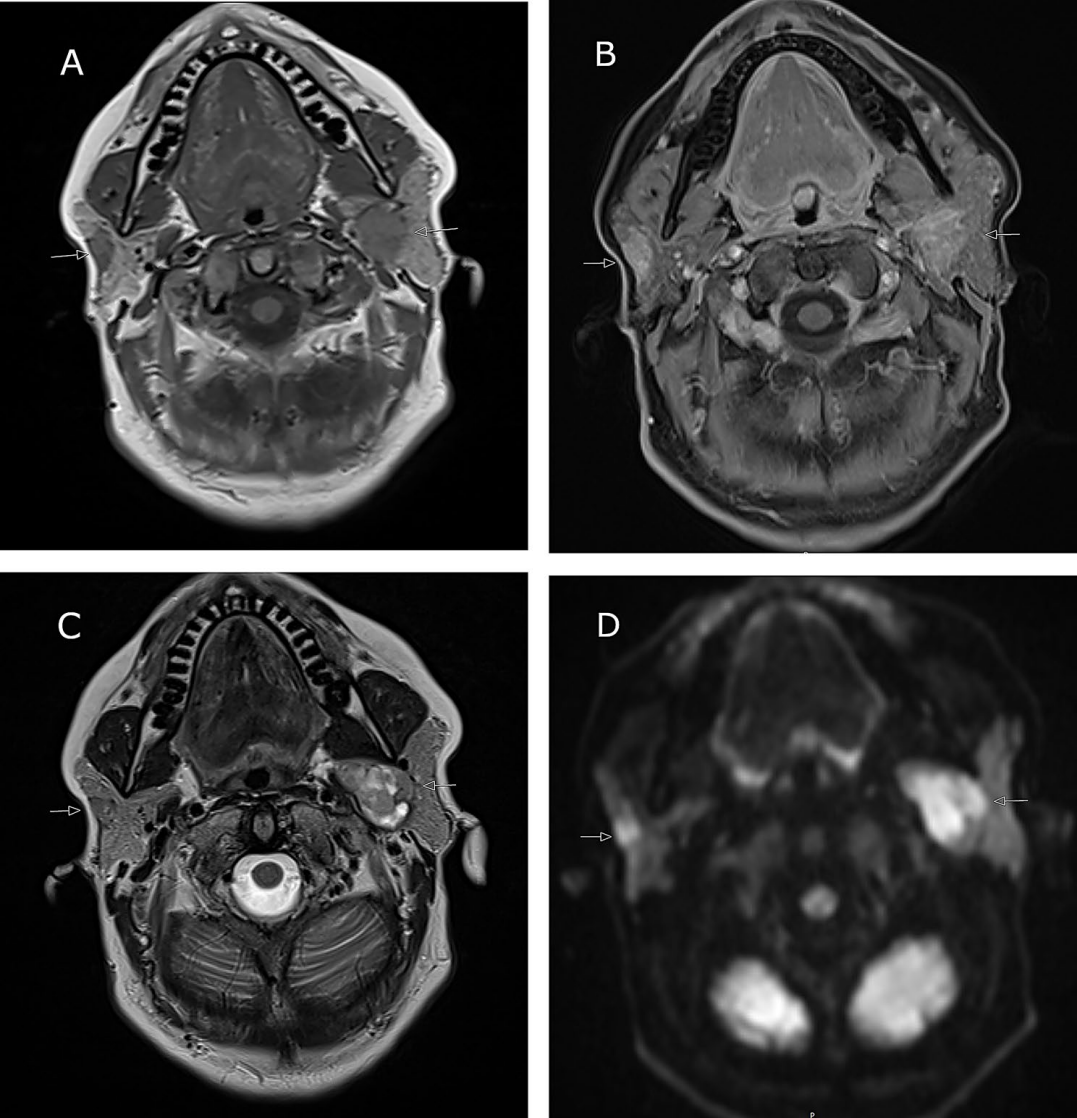
Patient 3, MR image of bilateral salivary gland tumors in a 51-year old female. **A**-**D** Bilateral intraparotid lesions with irregular borders and similar textures on the left side in the deep lobe and on the right side in the superficial lobe (arrows). **A**) Low signal on T1W, **B**) heterogeneous contrast enhancement on fat-suppressed TIW, **C**) mixed signal on T2W and **D**) restricted diffusion on DWI; the ADC was bilaterally approximately 1,1 × 10^− 3^ mm^2^/s (not shown). **E**) MR examination 15 months after surgery reveals a small subcutaneous recurrence (arrow) on the right side (fat-suppressed T1W with contrast enhancement)

MRI revealed bilateral parotid masses in the left deep lobe (3,8 cm) and the right superficial lobe (2,3 cm). The lesions were similar in appearance with well-demarcated, irregular borders and mixed signals on T2W images. Contrast uptake was heterogeneous, and diffusion was restricted on both sides (Fig. 5). No pathological lymph nodes were present.

FNAC was performed on the parotid gland on the left side, showing a cell-rich lesion consisting of groups of epithelial cells with granular cytoplasm and monotonous nuclei and a low nucleus/cytoplasmic ratio. On morphological grounds there was a preference for the diagnosis of acinic cell carcinoma, with a secretory carcinoma as a second option. No material was available for immunohistochemical evaluation. FNAC on the right side showed the same morphology as the tumor on left side. Just a small amount of material was left for immunohistochemical evaluation, showing positivity for DOG-1 and SOX10, which confirmed the diagnosis of a bilateral acinic cell carcinoma.

Both parotid glands were removed in 2 separate sessions as the patient requested surgery over two consecutive weeks due to fear of the simultaneous onset of bilateral facial paralysis. On the left side a subtotal parotidectomy was performed with inclusion of one buccal nerve branch and on the right side, ordinary superficial parotidectomy was performed.

**Fig. 6:**
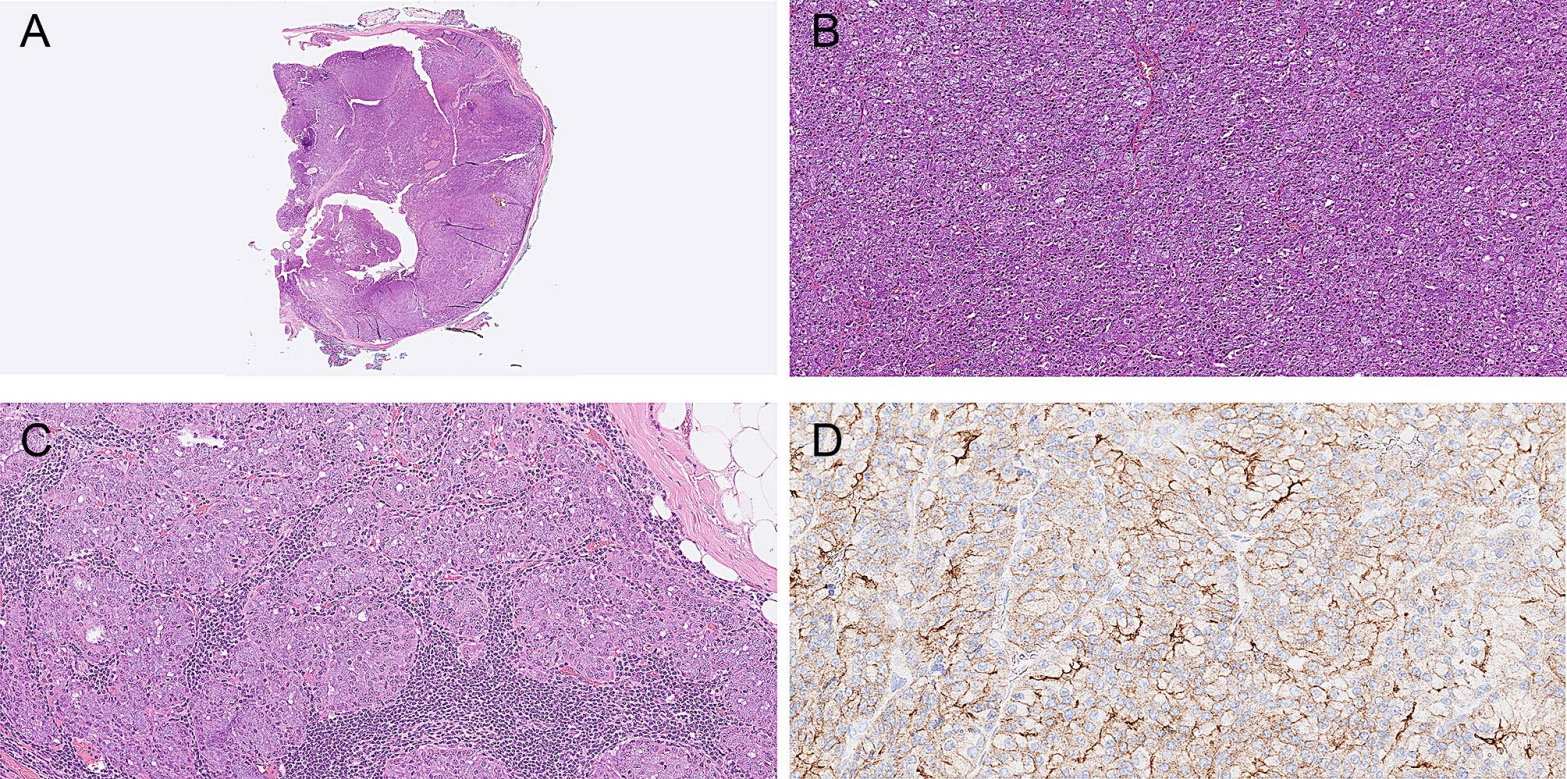
Patient 3, **A** and **B**. Overview and detail of acinic cell carcinoma on the left side, H&E;. **C**. Growth in the parotid lymph node on the right side. **D**. DOG-1 staining Both acinic cell carcinomas had the same morphology

The macroscopic and microscopic images of both tumors revealed the same findings (Fig. 6). Macroscopy revealed demarcated tumors without macroscopically visible invasion. Microscopy revealed mainly encapsulated, lobulated tumors consisting of cells with a large amount of granular cytoplasm and small, irregular nuclei, arranged in acinar structures. The lobules were separated by thick fibrous bands and spread microcysts were present. Focally, invasion in and through the tumor capsule into adjacent salivary gland tissue was observed. In and surrounding the tumors was some lymphoid tissue present. In the left parotid tumor, there was focal suspicion of intravascular growth, and on the right-side tumor in an intraparotid lymph node was observed. Immunohistochemistry revealed that the (acinar) tumor cells were positive for DOG-1 (cytoplasmic) but negative for S100 and GATA-3. The proliferation rate varied within the tumor, with 3–19%, on the right side and 7–40% on the left side. For both tumors the on FNAC suggested diagnosis acinic cell carcinoma was confirmed.

The patient was treated with postoperative radiotherapy on the left side. The same follow up as for patient 1 and 2 was applied. Fifteen months postoperatively a small recurrence appeared in the subcutaneous fat on the right side, corresponding to level 2 A lymph node region (Fig. 5E). On MRI the lesion seen could be in agreement with a small lymph node. However, based on its location, close to the previous tumor, and growth, a recurrence was suspected. A selective neck dissection including levels 2 to 5 was therefore performed on the right side, together with a small amount of overlying skin at level 2 A. Histopathology revealed a radically removed subcutaneously located lesion of the previously diagnosed acinic cell carcinoma with a proliferation rate of 40–50%, pointing to high grade transformation of the tumor. All the lymph nodes were tumor free. No tumor occurred during recent follow-up.

## Discussion

Although the incidence of (uni- or bilateral) synchronous and metachronous salivary gland tumors is rare it is important to think of the possibility during diagnostic work-up of a patient with a salivary gland tumor in order to early diagnose and treat these tumors [1].

The majority of bilateral malignant salivary gland neoplasms previously reported are acinic cell carcinomas [6, 8, 9]; other reported bilateral malignancies are adenocarcinomas, mucoepidermoid carcinomas and epithelial-myoepithelial carcinomas.

Salivary gland tumors can occur synchronously, metachronously, and ipsi- or bilaterally. All tumors in the patients in our study occurred synchronously and bilaterally, two patients had the same histology on both sides. In Patient 2, a bilateral occurrence of an epithelial-myoepithelial carcinoma was considered due to the biphasic architecture of the tumor. However, on the basis of the histomorphology and the immunohistochemical and molecular findings a final diagnosis of a bilateral pleomorphic adenoma was made.

The group of multiple tumors with different histologies is significantly smaller, comprising less than 0.3% of all tumors. In our study one of the 3 patients had a different histology in each parotid gland. In the case of synchronous tumors with different histologies a hybrid tumor, defined as a lesion composed of two or more distinct tumor types (benign or malignant) arising in the same topographical area, must be considered [5]. The most common combination of bilateral tumors with different histologies is Warthin tumors and pleomorphic adenomas, accounting for up to 42% of synchronous tumors [11]. Warthin tumors and pleomorphic adenomas can also occur together with other carcinomas of the salivary glands, such as Warthin tumor and secretory carcinoma, mucoepidermoid carcinoma or carcinoma ex pleomorphic adenoma in the same gland [12–14]. Even a combination of 3 histological types within one salivary gland was reported by Tanaka in 2006, with the malignant tumor being a salivary duct carcinoma [11]. Salivary duct carcinomas, which are part of multiple salivary gland tumors are very rare, with only a few cases reported [9, 15]. The diagnostic radiological features of our patient already revealed that the two tumors had different morphologies, which was confirmed histologically. Restricted diffusion and Type B curves indicate high cellularity, such as in highly cellular pleomorphic adenomas or malignancies. This likely contributes to the increased proliferation rate, as visualized histopathologically.

Notable is the simultaneous occurrence of salivary gland tumors with other oral tumors or extraglandular tumors, especially thyroid carcinomas and breast carcinomas [5]. We did not include these patients in this report.

The incidence of synchronous and metachronous salivary gland tumors has increased in the past decades because of improvements in tumor diagnostics, especially radiological techniques [2, 7]. Incidental synchronous (uni- or bilateral) salivary gland tumors have been found during radiological diagnostics for other salivary gland or nonsalivary gland tumors. The clinical, radiological and prognostic aspects of the different synchronous or metachronous benign and malignant salivary gland tumors do not differ from their non-synchronous/metachronous counterparts. The prognosis of the patient depends on the prognosis of the individual tumor with the worst morphology [2, 13].

All patients in this small series were women. No general male or female preference has been described in the literature for bilateral occurrence. The sex preference might be related to the specific histological diagnosis of the tumor. For example, acinic cell carcinomas seem to be predominant in females, especially in the younger age group [6]. On the other hand, Warthin tumors are more commonly observed in male patients [13].

The role of FNAC in salivary gland tumors is, in many institutions, the first choice for obtaining a diagnosis before treatment. The sensitivity and specificity of FNAC in differentiating between benign and malignant pathologies still varies significantly across studies [12, 13]. However, recent large studies have shown a sensitivity and specificity, which demonstrates increasing diagnostic utility [16, 17]. In our patients, FNAC helped to identify the lesions as tumors and, in most instances, a diagnosis that led to surgery for definitive diagnosis.

The treatment of choice for any diagnosis is complete surgical resection with adequate margins and, depending on histology, adjuvant treatment can be given [14]. In our patients, treatment consisted of complete surgical excision. In Patient 1, whose salivary duct carcinoma was the first tumor to be diagnosed, a neck dissection was performed and postoperative radiotherapy given. The pleomorphic adenoma on the contralateral site was an incidental finding, and the tumor removed for histological diagnosis and curative treatment.

Warthin tumors are the most common bilateral salivary gland tumors [18]. Currently, with the advanced radiological techniques, combined with FNAC, a diagnosis of bilateral Warthin tumors can often be made. Because of their usual slow growth, minimal clinical symptoms, and the advanced age of patients, active surveillance has become more widely the first approach. This might be the reason that in the short time-scale of this report, no bilateral Warthin tumors were found [19].

Different theories about the origin of multiple salivary gland tumors have been proposed. In case of a pleomorphic adenoma, different options have been suggested, such as multiple tumor foci or a metastasizing pleomorphic adenoma [4, 20]. A metastasizing pleomorphic adenoma is a very rare tumor, which should be diagnosed after thorough diagnostic work-up, excluding the possibility of a synchronous or metachronous tumor or a recurrence, as was shown by Alshagroud [20]. Hattori reported that it is not reasonable, in case of synchronous tumors, to consider the parotid gland tumor on one side to be a metastasis from the other side, as lymphatic drainage and blood supply have no direct connection between the two glands [10]. This may also be attributed to the fact that the majority of reported malignant bilateral parotid gland tumors do not metastasize to the lymph nodes or other organs.

Radiation has been suggested as an etiological factor, especially after nuclear accidents [1, 21]. Smoking might be important in the etiology of bilateral Warthin tumors. Possible genetic susceptibility has also been suggested for bilateral parotid pleomorphic adenoma. Ahn et al. describe the familial occurrence of bilateral synchronous pleomorphic adenomas of the parotid with the presence of a chromosomal translocation of t (3; 12) (p21; q15) [22]. Chromosome 12q breakpoints have previously been described in pleomorphic adenomas of the parotid gland and a variety of other solid neoplasms. Ahn suggests that breakpoints on the long arm of chromosome 12 seem to affect a growth regulatory process allowing these tumors to arise. No specific genes involved in this process were mentioned. However, a recent study by Stenman et al. mentions involvement of HMGA2 and MDM2 genes in individual pleomorphic adenomas with 12q13-15 aberrations [23]. Whether these genes also play a specific role in multiple tumors is not known so far.

In none of our cases an identifiable etiological factor could, however, be identified.

In conclusion, our cases and the literature review highlight the importance of an accurate clinical and radiological head and neck evaluation prior to surgery, including evaluation of other salivary glands to detect synchronous salivary gland tumors. In our opinion, MRI is mandatory to early diagnose multiple or contralateral tumors. Besides, a thorough follow-up is necessary to detect metachronous salivary gland tumors and recurrences or metatastic disease. The recommended treatment for bilateral tumors is the surgical approach similar to that indicated for solitary tumors.

## Data Availability

No datasets were generated or analysed during the current study.
